# Mechanical and Physical Changes in Bio-Polybutylene-Succinate Induced by UVC Ray Photodegradation

**DOI:** 10.3390/polym16091288

**Published:** 2024-05-04

**Authors:** Cristina Scolaro, Salim Brahimi, Aurora Falcone, Valentina Beghetto, Annamaria Visco

**Affiliations:** 1 Department of Engineering, University of Messina, C. da Di Dio, 98166 Messina, Italy; salim.brahimi@studenti.unime.it (S.B.); aurora.falcone@studenti.unime.it (A.F.); 2 Crossing S.r.l., Viale della Repubblica 193/b, 31100 Treviso, Italy; valentina.beghetto@crossing-srl.com; 3 Department of Molecular Sciences and Nanosystems, University Ca’ Foscari of Venice, Via Torino5 155, 30172 Mestre, Italy; 4 Consorzio Interuniversitario per le Reattività Chimiche e la Catalisi (CIRCC), Via C. Ulpiani 27, 70126 Bari, Italy; 5 Institute for Polymers, Composites and Biomaterials—CNR IPCB, Via Paolo Gaifami 18, 95126 Catania, Italy

**Keywords:** bioplastics, UVC rays, photodegradation, mechanical test, surface characterization

## Abstract

Bio-polybutylene succinate (PBS) is a biodegradable polymer obtained from renewable feedstock having physical–mechanical properties like traditional low-density polyethylene (LDPE). PBS is employed by many manufacturing sectors, from biomedical to agri-food and cosmetics. Although some studies have already evaluated the resistance of PBS to photodegradation caused by natural outdoor solar exposure (UVA-UVB), a systematic study on the resistance to degradation caused by exposure to UVC rays, which is the subject of this study, has not yet been carried out. PBS was exposed to UVC either neat or filled with 2% carbon black (CB). Mechanical and physical characterization (tensile, hardness, calorimetry, contact angle, morphology, and surface roughness analyses) indicates that the bulk and surface properties of the polymer matrix changes after exposure to UVC radiations, due to a severe degradation. However, the presence of carbon black compensates for the degradation phenomenon. Because UVC rays are used for the sterilization process, necessary in applications such as biomedical, cosmetic, pharmaceutical, food, and other products, a comparison of the protocol used in this paper with the literature’s data has been reported and discussed.

## 1. Introduction

Biodegradable and Bio-based Polymers (BBPs) from renewable resources are an interesting alternative to traditional Fossil-based Polymers (FbPs), due to their easy biodegradation in the soil compared to FbPs which degrade in thousands of years [1,2,3]. This feature is crucial to eliminate the serious problem of FbP pollution, for the protection of ecosystems and the health of the entire planet [4,5].

Bio-polybutylene succinate (PBS) is an example of both a bio-based and biodegradable polymer obtained from renewable sources. PBS is an alternative to commercial fossil-based plastics due to its interesting physical–chemical properties, which are similar to those of low-density polyethylene (LDPE) [6]; low production costs; and the possibility of obtaining both succinic acid and 1,4-butanediol from renewable sources (this makes PBS a completely bio-based polymer) [7]. PBS is a white, semi-crystalline, ductile, and thermoplastic polymer with a density of 1.25 g/cm^3^, a melting point (T_m_) between 90 and 120 °C, and a glass transition temperature (T_g_) ranging from about −45 °C to −10 °C [8]. Its high extrusion speed and short molding cycles make it suitable for multiple forming processes [9]. Over the last ten years, numerous authors have studied PBS in the literature, highlighting its multiple properties such as biodegradability, good compatibility with other biopolymers, easy printability, excellent food grade, good mechanical and thermal properties, resistance to heat, and resistance to chemical agents [10,11,12].

PBS finds application in various sectors, such as fishing (for the fabrication of fishing nets); textiles (sheets of non-woven fabric); agricultural fields; manufacturing mulching films and sheets of packaging both for food and for cosmetics’ packaging; cooking (disposable products such as crockery); and biomedical fields (scaffolds for tissue engineering, micro- and nanoparticles for drug delivery, wound dressings, 3D and 4D printing, electrospinning processing) [10,13,14,15,16].

The biodegradability and compostability of PBS have been deeply studied in the literature [17,18,19,20]. On the contrary, an aspect which has not yet been widely studied in the literature is its stability against UV rays. The effects of photoaging and photodegradation represent an important problem for the life of polymers, both for outdoor applications as mentioned above and for sterilization processes [21,22,23].

Only very few authors have dealt with this topic for PBS, unlike for widespread plastics such as PE. Fritz et al. [6] investigated the photoaging effects on PBS surfaces only under UVA and UVB rays for outdoor applications like packaging, agriculture, or plumbing, and the data achieved were compared to those of HDPE.

Nevertheless, it should be considered that there are three types of UV radiation: UV-A (315–390 nm), UV-B (280–315 nm), and UV-C (100–280 nm). While UV-A and UV-B radiation are produced by the sun and naturally reach the earth’s troposphere, UV-C radiation is created artificially and is employed for sterilization [24].

The effects of UV light (UVA-UVB) photo-oxidation on FbPs are well known and thoroughly studied, showing a decrease in hydrophobicity and tensile strength favoring degradation. Moreover, changes in the structure (polymer branching, crystallinity, hydrophilicity), and surface morphology have also been evidenced [25,26].

The sterilizability of BBPs with UVC is of particular interest following the recently concluded pandemic [24,27,28]. UVC rays can affect microorganisms and plants, damaging the DNA of bacteria and viruses. At the same time, UVC irradiation is also responsible for the decomposition and degradation of many organic compounds (such as polymers), as well as the loss of the gloss and mechanical properties of plastics. Nevertheless, in many cases (such as, for example, biomedical, medical, food, drink, pharma, and cosmetic applications) manufacturing is subjected to sterilization processes before use; therefore, the influence of UVC on polymers is of crucial importance.

To prevent polymer degradation, inorganic nanoparticles, such as alumina, silica, and titanium dioxide, may be added as UV inhibitors [29]. Cai et al. [30] in their work reported the use of titanium dioxide in PBSA (polyethylene succinate-co-butylene adipate) films as a barrier to UVA and UVB rays. In fact, thanks to its excellent absorption, scattering, and reflection capacity towards UV rays, TiO_2_ has proven to be particularly effective. Furthermore, the white color does not alter the aesthetic properties of bioplastics.

To the best of our knowledge, there are no previous works in the literature reporting the influence on the physical–mechanical properties of PBS because of UVC irradiation. Thus, in this work the resistance to UVC of PBS and PBS stabilized with UV adsorbers (carbon black—CB, at 2% by weight) have been studied. The use of carbon black (CB) to PBS has been previously reported in the literature, albeit to enhance its electrical and mechanical properties [31,32] or antimicrobial features [33]. The blackening caused by CB should be particularly effective with UVC rays, which have higher energy than UVA and UVB. Samples of pure PBS with 2 wt.% CS were characterized by hardness, tensile, calorimetric, wettability, roughness, and morphological tests before and after UVC irradiation [28,34]. Monitoring both the modification of the surface and of PBS in bulk, the degradation induced by the absorption of UVC rays was monitored and the effectiveness of the presence of CB in this bioplastic analyzed.

Since UVC is commonly employed for surface sterilization, a comparison of the protocol employed in this work with the literature’s data is also reported.

## 2. Materials and Methods

### 2.1. Materials Employed

Bio-PBS FZ71-(PBS) was purchased from MITSUBISHI Chemical Performance Polymers Inc. supplier. Carbon black (CB) was purchased from CABOT Chemical Corporation, having a density of 264 kg/m^3^ and an average particle size of 30 nm. CB is used as a pigment, conductive filler material, particulate reinforcement, and ultraviolet light (UV) absorber [35]. All materials were pre-dried at 60 °C for 4 h in an oven before processing.

The blend of PBS-CB was prepared from a direct melting process in a melt mixer machine, according to a previously reported protocol [31]. PBS, pure and with 2 wt.% of CB, was mixed in a Brabender Plasticorder PL2100 chamber at 140 °C, speed 40 rpm, for ten minutes.

**Figure 1:**
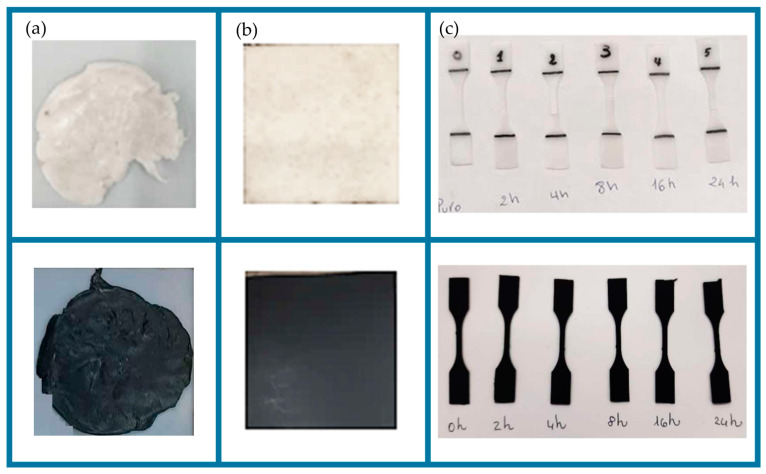
Blend (**a**) and sheets (**b**) of PBS and PBS-CB. Dog bones of PBS and PBS-CB at different UVC ray exposure times (0–24 h) (**c**).

The resulting blends (Figure 1a) were thermoformed in a uniaxial hot press (PM 20-200, supplied by DGTS s.r.l., Veduggio Con Colzano (MB), Italy) at 140 °C for 15 min, at a pressure of 100 bar to obtain 6 × 6 cm square sheets, with 1 mm thickness (Figure 1b). Dog-bone samples were obtained by a Ray-Ran cutter machine according to ASTM D638 M-3 (Figure 1c). According to ASTM mentioned before, the sample’s dog-bone-shape was as follows: total sample length = 60 mm, width = 10 mm, useful section width = 2.5 mm, sample thickness = 1 mm, clamping section length = 25 mm.

### 2.2. Irradiation Set-Up

**Figure 2:**
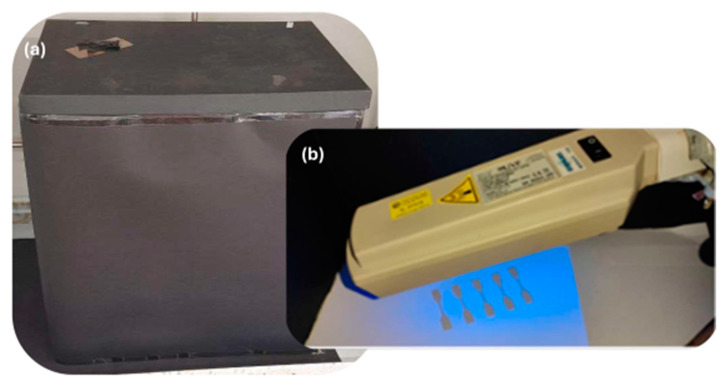
Black chamber (**a**) in which the dog-bone-shape samples are exposed to UVC rays and (**b**) dog bones of PBS and PBS-CB under UVC lamp.

UVC ray exposure conditions were in agreement with Amza et al. [28,34]. Dog-bone-shape samples, with a thickness of 1 mm, were exposed in a black chamber to UVC rays (UVP lamp mod. UVG-54 handheld, 254 nm, 230 V/6 W, 2200 μW at 7.62 cm, LLC, Upland, CA, USA) for 2-4-8-16-24 h (Figure 1c and Figure 2a,b) at room temperature of 25 °C and relative humidity (RH) of 21% [21,36]. The distance from the lamp to the samples was 15 cm and was kept constant during all treatments. The UVC exposure of all samples was replicated twice, on each side of the sample. The UVC average intensity was 860 μW·cm^−2^ ± 10 μW·cm^−2^ at the dog-bone surface (Blak-Ray model J-225 Ultraviolet Meter). The treatment doses ranged from ~6.2 to 74.3 J/cm^−2^ (intensity per time). The values of the different exposure times are presented in Table 1.

Unlike for UVA and UVB rays, standard regulations on the use of UV-C tests are not well defined. An appendix of ISO 4892-2 considers the use of 254 nm mercury lamp with an intensity exposure of 10 W/m^2^; the BIFMA (Business and Institutional Furniture Manufacturers Association) for healthcare devices considers an exposure dose lamp of 291 KJ/m^2^, a temperature of 50 °C, and exposure times between 12 and 24 h [28,34].

### 2.3. Mechanical Characterization Tests

Shore D hardness was used to measure the hardness of PBS and PBS-CB before and after UVC ray exposure, according to the ASTM D-2240 standard using a Shore D durometer model PCE TH210FJ from PCE Italia s.r.l., Capannori (LU), Italy (reading precision of 0.1 Shore D units and precision of ±1 degrees, in the scale range from 0 to 100). Above the durometer there was a weight of 5 kg which was used to penetrate the head. Every test was repeated on the surface of ten pieces of bioplastic sheet, with 1 mm thickness, of different samples for each formulation, and average values calculated.

Tensile tests were performed on PBS and PBS-CB dog bones according to the ASTM D 638-03 standard with a Lloyd LR10K Universal Dynamometer machine (load cell 0.5 kN, preload 1.00 N, speed 2 mm/min) purchased from Elis–Electronic Instruments & Systems S.r.l., Rome, Italy. Tests were carried out at 25 °C and relative humidity (RH) of 21%.

Mechanical parameters, such as Young’s modulus (E [MPa]), stress at break (σ_r_ [MPa]), deformation at break (ε_r_ [%]), and work at break (W_r_ [J]), were obtained as the result of the average values obtained from eight samples (for each type). The breaking point is considered as the stress value at the maximum possible deformation of the sample at which it breaks.

### 2.4. Physical Characterization Tests

Calorimetric DSC analyses and surface hardness, wettability, roughness, and mechanical tests were performed on pieces of bioplastic sheet with 1 mm thickness (weight about 6–10 mg) before and after UVC exposure. Differential scanning calorimetry (DSC) was performed under nitrogen with a flow rate of 50 mL min^−1^, from room temperature to 200 °C, with a heating rate of 10 °C min^−1^ and water cooling by a TAQ500 instrument (TA Instruments, New Castle, DE, USA). The percentage crystallinity degree (*X_c_*), according to ASTM D 3417-99, was calculated by Equation (1):

(1) $$X_{c}\left(\%\right)=\left(\frac{{\Delta H}_{m}}{{\Delta H}_{0}\;\cdot \;\left(\frac{1-w}{100}\right)}\right)\;\cdot \;100$$

where ΔH_m_, and ΔH_0_ are the experimental melting enthalpy, and the theoretical heat of fusion of 100% crystalline PBS (ΔH_0_ = 110.3 J/g), respectively, and *w* is the weight fraction of CB in the formulation. All measurements were carried out in triplicate [37,38].

Sample’s surface roughness (*Ra*) was calculated by a roughness tester Surftest SJ-210- Series 178 (Mitutoyo S.r.l., Milan, Italy) by Equation (2):

(2) $$Ra=\frac{1}{N}\overset{n}{\underset{i=1}{\sum }}\left|Yi\right|$$

where *Ra* represents the arithmetic mean of the absolute values of the deviations of the evaluation profile (*Yi*) from the mean line.

Contact angle *θ* was evaluated by the *θ*/2 method (KYOWA—DMs-401) which measures the contact angle “theta” (*θ*) of a 2 μL drop of liquid (deionized water, or human blood) on the horizontal surface of the sample [39], according to ASTM D 7334 and Equations (3) and (4):

(3) $$\;\theta_{w}=2\mathrm{arctg}\left(\frac{2h}{d}\right)$$

(4) $$\theta_{Y}=arcos\;\left(\frac{cos\theta_{w}}{r}\right)$$

where *d* is the diameter (mm) and *h* is the height (mm) of the drop, *θ_w_* the Wenzel angle, *r* is the surface roughness, and *θ_Y_* is the Young contact angle of equilibrium on perfectly smooth surface.

By also considering the possible biomedical applications of PBS, the wettability of a biological fluid like human blood was tested. Human blood was extracted from a healthy adult volunteer in a clinical laboratory of biological analyses and immediately stored in an original sterile tube (BD Vacutainer K3E) with 5.4 mg ethylenediaminetetraacetic acid (EDTA) at a temperature between 2 °C and 6 °C. The EDTA solution inhibits the coagulation cascade, preserving the blood [40].

UVC-induced surface changes on PBS and PBS-CB surfaces were observed by scanning electron microscopy (SEM) performed by a ZEISS Crossbeam 540 microscope (Carl Zeiss Microscopy GmbH, 07745 Jena, Germany), at an accelerating voltage of 5 KV and magnification 8000×. Before carrying out the SEM analysis, all samples were coated with a thin layer of chromium using a Quorum Q 150T-ES coating machine (Quorum Technologies Limited Company, West Sussex RH19 2HL, UK) to make them electrically conductive.

Prism 8.0.2 statistical software (GraphPad, Inc., La Jolla, CA, USA) was used for the statistical analysis. Data are reported as mean ± SD (±Standard Deviation) at a significance level of *p* < 0.05. The Shapiro–Wilk test was used for normality and lognormality tests of data, and Brown–Forsythe test for homogeneity of the variance test. Since all data used in this study satisfied these two tests, two-way analysis of variance (ANOVA) with Bonferroni’s post hoc test was performed to evaluate the statistical significance of the differences between the groups (significance level: 0.05).

## 3. Results and Discussion

### 3.1. Mechanical Characterization Tests

The resistance to UVC rays of PBS and PBS-CB was checked through hardness and mechanical tensile tests.

The Shore D hardness values (HD) of PBS and PBS-CB versus the UVC exposure time are listed in Table 1. The hardness of PBS before UVC exposure (t = 0 h) is 68.6 HD, and it slightly grows after the addition of carbon black (69.1 HD), reasonably due to the presence of CB that is harder than PBS [41].

**Figure 3:**
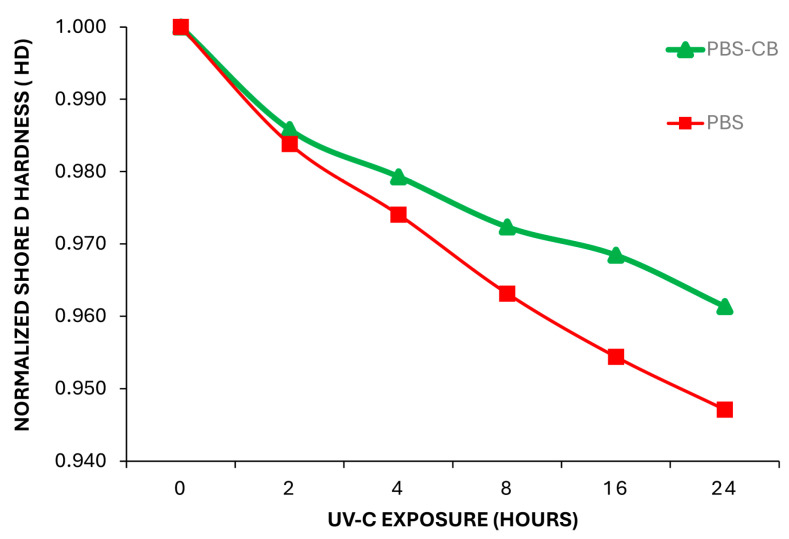
Shore D hardness of PBS and PBS-CB during the UVC ray exposure.

The Shore D hardness progressively decreases with the exposure time in both PBS and PBS-CB, demonstrating that the polymeric surface is sensitive to UVC ray action. Although the hardness of both PBS and PBS-CB is equivalent after 2 h of UVC exposure, the hardness of PBS-CB decreases faster (from 69.1 HD to 66.4 HD, ΔHD = −3.6) after longer exposure times (up to 24 h) compared to that of PBS (from 68.6 HD to 65.0 HD, ΔHD = −2.7 HD). This is shown in Figure 3, where the normalized shore D hardness values are plotted versus the UVC ray exposure time. These data suggest that the presence of CB acts as a barrier against UVC rays for PBS polymer, decreasing the degradation effects on PBS. This finding is in agreement with the study conducted by Liu et al. [35] which demonstrated that carbon black (between 1.5–3.0 wt.%) was able to stabilize LLDPE films under UVA/UVB rays.

**Figure 4:**
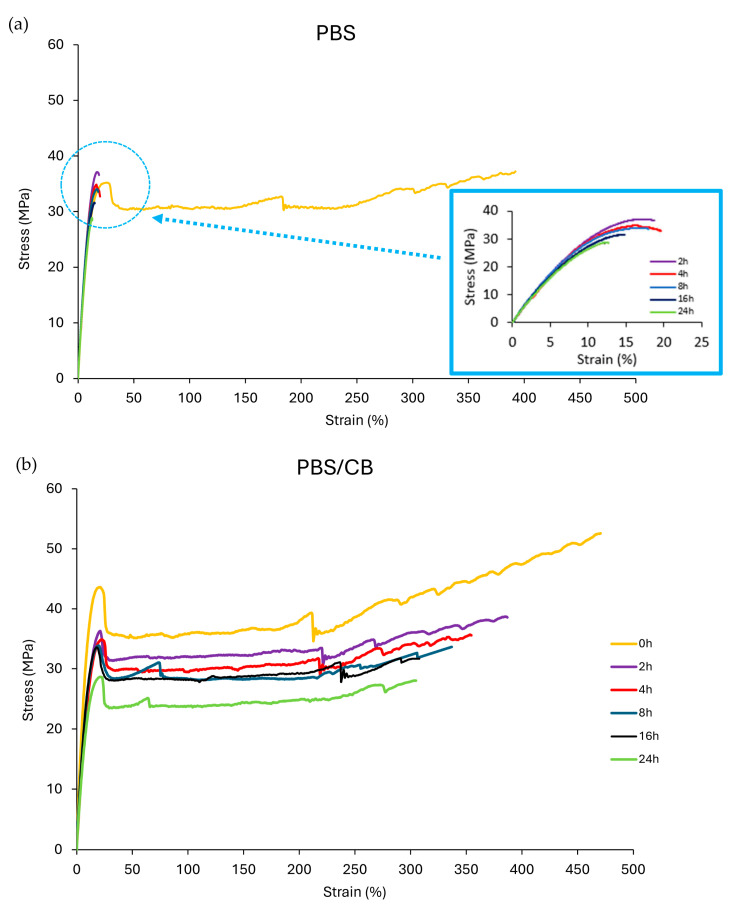
Stress–strain curves of PBS (**a**) and PBS-CB (**b**) during the UVC ray exposure.

The stabilization of PBS against UVC due to the presence of CB is even more evident in bulk mechanical tests as evidenced by stress–strain profiles reported in Figure 4 and data in Table 2. UVC exposure has notable damaging effects, so PBS transitions from a ductile and highly deformable material into a brittle polymer (see Figure 4a and Table 2). Indeed, the deformation at break (ε) of PBS is 411.61% (*p* < 0.0001), and it reduces to 18.75% after 2 h of UVC exposure (*p* < 0.0001), lowering by more than one magnitude order with respect to that of un-irradiated PBS. The deformation after 24 h did not significantly reduce compared to the data achieved after 2 h: 12% (*p* < 0.0001) compared to 18%, as shown in Table 2.

As can be seen from the data in Table 2, the initial yield strength (σ_y_) of PBS disappears completely after just 2 h of irradiation. On the contrary, PBS-CB always displays yielding during all 24 h of exposure to UVC rays (Table 2 and Figure 4b) showing that the ductility of PBS-CB is maintained even after 24 h of irradiation (*p* < 0.0001). The elongation at break in unirradiated PBS-CB (t = 0 h) is 470%, and it reduces to 300% after 24 h (*p* < 0.0001). The preservation of the ductility of PBS-CB after 24 h of UVC exposure (*p* < 0.0001) indicates the effectiveness of CB as an UVC adsorber. Overall, both Shore D hardness and tensile mechanical performance are less influenced in PBS-CB than pure PBS consequent to UVC irradiation.

Amza et al. carried out UVC exposure tests on PLA (poly-lactic acid) and PETG (polyethylene terephthalate glycol), both used for 3D printing [28]. The authors observed a decrease in mechanical properties in PLA, which was even more so in PETG. Additionally, Lin et al. [26] highlighted that the excessive exposure of microplastics lost at sea to UV rays (such as PS-polystyrene, PVC polyvinyl chloride, and PET-polyethylene terephthalate) significantly altered their morphological, chemical, and physical properties.

To verify physical property changes, as highlighted by the authors above, tests on calorimetric behavior and surface features were performed and are discussed in the following section.

### 3.2. Physical Characterization Tests

**Figure 5:**
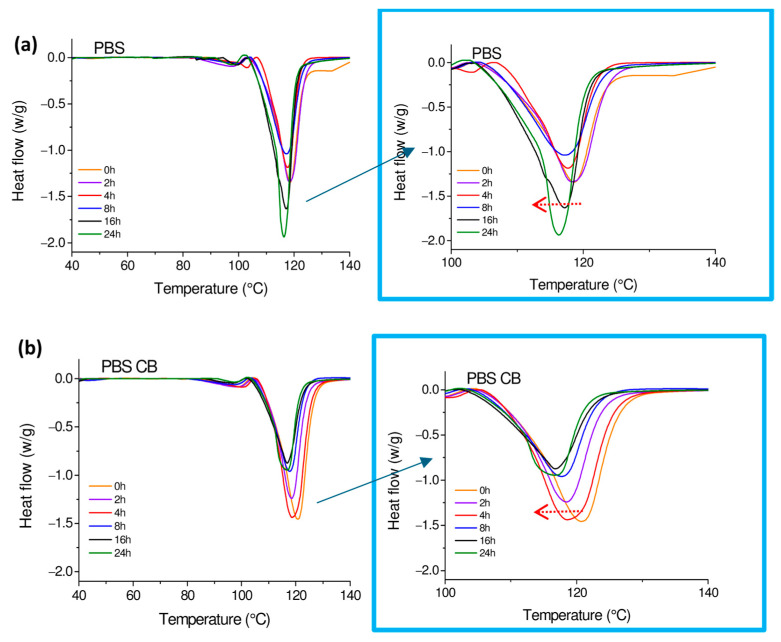
DSC curves of PBS (**a**) and PBS-CB (**b**) vs. the UVC exposure time.

Melting peaks obtained by DSC analysis of PBS and PBS-CB during the 24 h UVC irradiation experiments are reported in Figure 5a,b, while melting temperature, enthalpy of melting, and crystalline degree values are listed in Table 3. Melting temperature decreased in both PBS and PBS-CB during UVC exposure, showing a slight decrease in temperature stability, although values measured in PBS-CB were slightly higher than those in PBS at the same irradiation time. The melting temperature of PBS increases with the increase in molecular weight [42]. Therefore, the decrease in melting temperature values during irradiation suggests that the polymeric matrix undergoes a degradation effect during exposure to UVC rays with a consequent decrease in its molecular weight.

**Figure 6:**
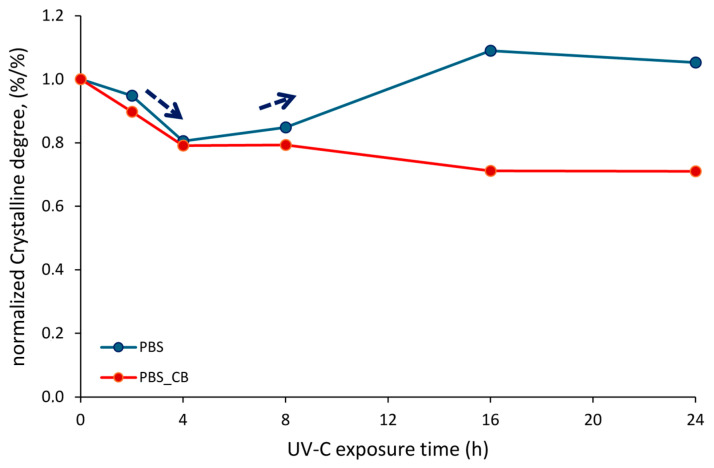
Normalized crystalline degree values of PBS and PBS-CB vs. the UVC exposure time.

Normalized crystalline degree values of PBS and PBS-CB versus the UVC exposure time are plotted in Figure 6. The trend in the degree of crystallinity in pure PBS is not uniform: a decrease in crystallinity is observed until 4 h of irradiation, followed by an increase due to longer exposure to UVC (see the arrows in the picture). This result was also observed by Ainali et al. [43] regarding polyethylene exposed to UV rays. In their study, the authors correlated the decrease and subsequent regrowth to the scission of the PE macromolecular chain and to the subsequent crystallization of unchained segments within the polymer matrix.

The crystallinity degree of PBS changed when CB was added to the polymer since the values were lower than those found in pure PBS, especially after prolonged exposure times (16 h and 24 h). This result could be explained by considering the protective action of CB, which inhibits chain scission and, consequently, the crystallization of unchained segments. To gain deeper insight in the chemical reactions and structural changes occurring during UVC irradiation in PBS and PBS-CB (as per GPC, FTIR, and rheological analyses) are ongoing to understand the changes induced by UVC exposure and to explain these phenomena [44].

Physical surface characterization analyses were further performed by means of roughness and wettability tests of two different fluids (distilled water and human blood) on PBS and PBS-CB.

**Figure 7:**
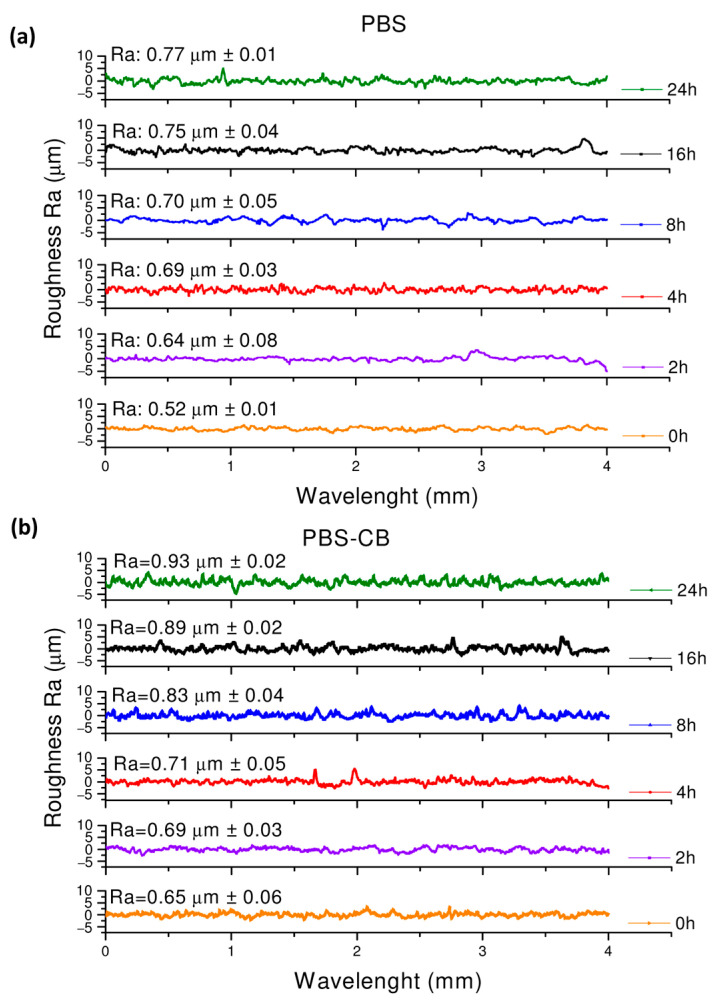
Roughness profiles of PBS (**a**) and of PBS-CB (**b**).

The roughness values of the PBS surface increased up to 48% due to UVC exposure (from 0.52 μm to 0.77 μm after 24 h, Figure 7a). In agreement with the literature, these data clearly show that UVC ray photodegradation induces defects and irregularities on the polymeric surface. In fact, Ainali et al. [43] and Rasouli et al. [45] showed that a rougher, damaged surface was obtained after UV exposure in polymers such as low-density and high-density PE, polypropylene (PP), and polystyrene (PS). The values of surface roughness of PBS-CB were generally higher compared to those of PBS at all UVC exposure times, reasonably due to the presence of carbon black particles. The roughness of PBS-CB increased up to 43% due to UVC exposure from 0.65 μm to 0.93 μm after 24 h (Figure 7b).

**Figure 8:**
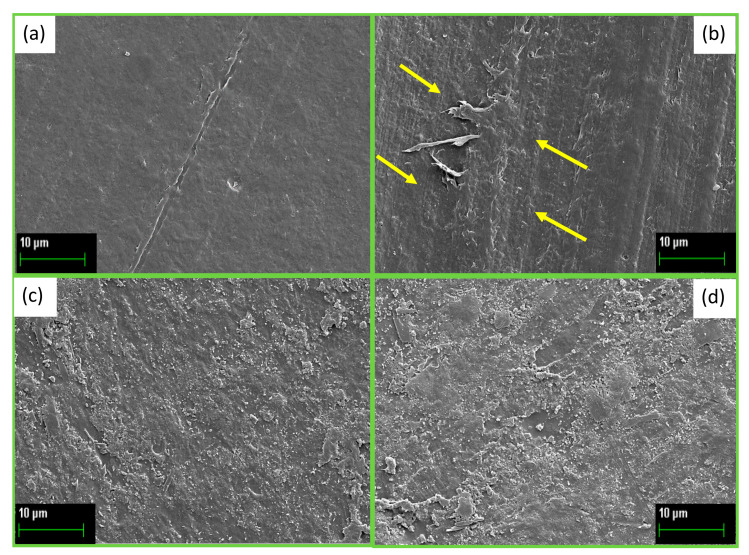
SEM microscopy images at magnification of 8000× of PBS and PBS-CB after 0 h (**a**,**c**, respectively) and after 24 h of UVC exposure (**b**,**d**, respectively).

To observe the effects induced on the surface of PBS and PBS CB during UV exposure, the surface morphology was analyzed using SEM microscopy analysis. SEM micrographs reported in Figure 8a,b were taken from pure PBS (0 h) and after 24 h of UVC exposure, respectively. Figure 8a shows the typical smooth surface of polymers. After 24 h of exposure, the surface alters, forming reliefs and filaments (see, for example, the yellow arrows in Figure 8b). This shows a surface modification effect resulting from UVC absorption by pure PBS. Instead, PBS-CB shows a rougher surface than that of pure PBS, due to the presence of carbon black (Figure 8c). The surface irregularities are maintained even after 24 h of UVC exposure (Figure 8d), but there are no appreciable differences compared to the non-irradiated PBS-CB sample. This confirms an effective anti-UVC protective action due to the presence of carbon black. All SEM optical images agree with the results of the surface roughness measurement.

**Figure 9:**
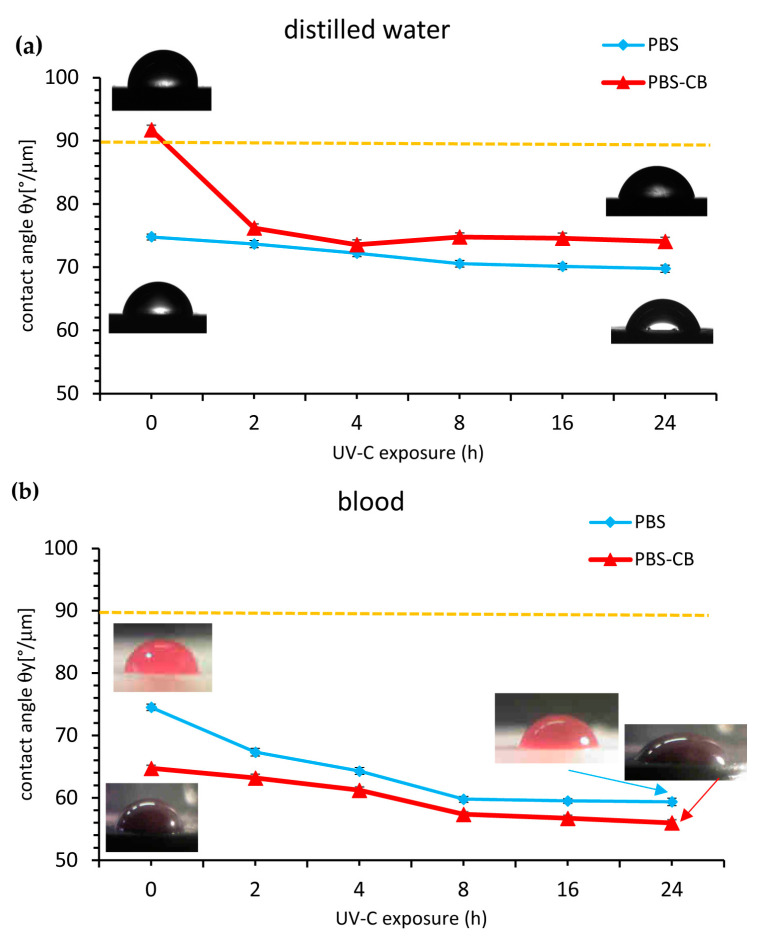
Young contact angle vs. UVC exposure of PBS and PBS-CB of water drops (**a**) and of blood drops (**b**).

Additionally, in Table 4 the Wenzel Teta values of all the samples tested are listed, and in Figure 9 the Young Teta values of water and of blood on PBS and on PBS-CB surfaces are plotted versus the irradiation time. Results indicate that the presence of CB in PBS, at time = 0 h, changes its affinity with distilled water because the theta contact angle increased from 82° to 91° (*p* < 0.0001), improving its hydrophobicity. On the contrary, the affinity of both PBS and PBS-CB was higher with blood, compared to that with water, since theta values are lower than 90° (82.1° and 73.9°, respectively; see Table 4). Consequent to UVC irradiation, the hydrophobicity of PBS decreased from 82.2° (0 h) to 74.6° (24 h), *p* < 0.0001, in agreement with the literature. In fact, Rasouli et al. [45] related the decrease in the contact angle in composites based on nano-sized zinc oxide (ZnO) and high-density polyethylene (HDPE) to changes in the surface chemical composition and to the increase in roughness caused by surface cracking. Lin et al. [26] highlighted changes in the contact angle occurring at different speeds depending on the chemical structure of the polymer. In this study, it has been observed that UV rays induce photolysis reactions with chain scission and the creation of new oxidized functional groups, such as carbonyl (C=O), and hydroxyl (C-OH), and carboxyl (CO-OH), which react with water through hydrogen bonds [26,46].

A different situation occurs when blood is employed. The presence of CB in PBS increased the affinity for blood (Figure 5b and Table 2) and the contact angle of blood tested on PBS (82.1°), with the sample surface being higher than that of PBS-CB (73.9°), *p* < 0.0001. Blood’s theta values of the PBS surface treated with UVC gradually decreased from 82.1° to 67.0° (*p* < 0.0001), while the PBS-CB theta values changed from 73.9° to 62.8° (*p* < 0.0001). This indicates a strong affinity of CB for blood (in agreement with the literature’s data [47]), which increases with the degradation induced in the bioplastic by UVC exposure. Thus, the addition of carbon black makes the surface of PBS slightly less hydrophilic and more blood-philic.

### 3.3. Literature Comparison of Operative Parameters Employed for UVC Irradiation of Polymers

The operative parameters employed in this work for the UVC irradiation of PBS were compared with those reported in the literature, with regard to the percentage of killed microorganisms (Table 5). From this comparison, it emerges that different irradiation conditions were used (exposure power, intensity, fluidity, time, and height, i.e., the distance from the lamp to the sample), which may influence the effectiveness of the irradiation, together with other factors including the following:

(i) Adsorption capacity as a function of material thickness.
(ii) Possible variations in the power of the UV source affecting the electromagnetic wavelength.
(iii) The presence of dust particles capable of protecting microorganisms from UV rays.
(iv) The ability of microorganisms to resist radiation during exposure.

Considering, for instance, the conditions employed by Bak et al. [48] (i.e., 6 W power, 0.4 × 10^−3^ W/cm^2^, intensity of 6 × 10^−3^ J/cm^2^ for times of 0.5 min up to 90 min), experiments carried out in this work were performed with equivalent UVC power (6 W), but at higher fluency (from 6.2 to 74.3 J/cm^2^) and longer exposure times (120–1440 min. or 2–24 h).

Since the UVC system should at least lead to a disinfection rate of 90% (corresponding to a reduction of 1-log), to achieve a decrease in bacteria up to 99.9% (corresponding to reduction of 3-log), a 200% increase in the exposure time or irradiance per square meter should be adopted [52]. Malayeri et al. [53] examined the fluence data (UV dose) required to achieve the log-incremental inactivation of bacteria, protozoa, viruses, and algae; the authors highlighted, as several studies have shown, that a fluence below 20 mJ/cm^2^ is sufficient to achieve a 90% kill rate (1-log reduction) for microorganisms, but it is sometimes necessary to increase the dose from 30 mJ/cm^2^ to 50 mJ/cm^2^ to kill viruses. The dose is further increased to 330 mJ/cm^2^ to kill mold and protozoan spores.

According to the literature’s data, it can be supposed that the intensity rate of 860 μJ/cm^2^ used in this work should determine a 90% killing rate (1-log reduction) of microorganisms after only 23 s of exposure (fluence dose of 20 mJ/cm^2^ = intensity × time). After 35–59 s (30/50 mJ/cm^2^), it should kill viruses and inactivate mold spores and protozoa after 384 s (6.4 min, 330 mJ/cm^2^).

## 4. Conclusions

Polybutylene succinate (PBS) represents an alternative to fossil-based plastics, since it possesses physical–mechanical characteristics similar to those of polyethylene, is also easily workable with the common technologies used for thermoplastics, and has the advantage of being bioderived and biodegradable.

In this study, the resistance of PBS to UVC exposure at room temperature was evaluated and compared to that of PBS prepared in the presence of 2% carbon black (CB). Although some studies had already evaluated the resistance of PBS to photodegradation caused by natural outdoor solar exposure (UVA-UVB), a systematic study on the degradation caused both on the surface and on the bulk by artificially induced UVC ray exposure had not yet been carried out.

Our study evidenced the changes in the bulk and surface properties of PBS exposed to UVC over a period of 24 h. UVC induced progressively severe damages in pure PBS. This effect is strongly reduced by the presence of CB, which absorbs UV rays and preserves the degradation of the bioplastic. Experimental results show a modification effect on both the surface and the bulk of the specimens tested. UVC rays degrade PBS due to photo-oxidation reactions. PBS loses its ductility and becomes noticeably brittle after only 2 h of exposure. The probable chain scission reactions in the amorphous part make PBS rougher and more hydrophilic.

The presence of 2 wt.% CB by weight of PBS proved effective in reducing the photodegradative effect since UVC rays are absorbed with a significant reduction in the degradation effects. The material, in fact, remains ductile even after 24 h of exposure to UVC rays. Although PBS-CB became increasingly hydrophilic with increasing exposure, it exhibited a slightly less hydrophilic character than that of pure PBS. The blood wettability trends of the PBS and PBS-CB samples during exposure to UVC were different: due to the intrinsic affinity of carbon black for blood, greater blood-philicity was observed in PBS-CB compared to in PBS. This phenomenon increases with increasing irradiation time.

UVC rays are used for the sterilization process, which is necessary in some applications such as in medicine, cosmetics, pharmaceuticals, foods, and other products. Since UVA-UVB rays are less powerful than UVC rays, a material that is well resistant to UVC rays can easily resist UVA-UVB rays.

A comparison with the literature’s data regarding the power of the UVC lamp employed in this work demonstrated that the conditions used here could induce accelerated aging and need to be appropriately remodulated in further studies, since under these conditions, PBS is very sensitive to photodegradation.

Additional studies will be devoted to optimizing exposure/dose times to kill different pathogens, define the optimal quantity by weight of CB in PBS, and compare the effect of carbon black with the effect of a white filler with less aesthetic impact (such as titanium dioxide) or others. Therefore, further future investigations will be repeated with much lower exposure times (seconds and/or minutes), which should be sufficient to kill all pathogens. These studies should be associated with a biological investigation of irradiated PBS and PBS-CB, to confirm the theoretical estimation of the reduction in pathogens.

## Figures and Tables

**Table 1:** Exposure and treatment doses (at 25 °C, RH = 21%, average intensity of 860 μW/cm^−2^) and Shore D values of PBS and PBS-CB during the UVC exposure time.

UVC Exposure Time (h)	Fluence Dose [J·cm^2^]	Shore D PBS (HD)	Shore D PBS-CB (HD)
0	-	68.6 ± 0.03	69.1 ± 0.03
2	6.2	67.5 ± 0.02	68.1 ± 0.05
4	12.8	66.8 ± 0.07	67.6 ± 0.01
8	24.8	66.1 ± 0.06	67.1 ± 0.03
16	49.6	65.5 ± 0.01	66.8 ± 0.03
24	74.3	65.0 ± 0.05	66.4 ± 0.07

**Table 2:** Tensile parameters of PBS and PBS-CB.

**UVC Exposure**	**Mechanical Parameter–Tensile Test of PBS**						
**Time** **(h)**	**E** **[MPa]**	**σ_y_** **[MPa]**	**ε_y_** **[%]**	**Load r** **[N]**	**σ_r_** **[MPa]**	**ε_r_** **[%]**	**L_r_** **[J]**
0	337.49 ± 4.71	35.31 ± 1.58	25.61 ± 1.33	88.90 ± 2.90	37.74 ± 2.23	411.61 ± 5.67	4.76 ± 0.09
2	346.11 ± 10.05	-	-	78.46 ± 5.03	37.44 ± 1.33	18.75 ± 1.67	0.15 ± 0.02
4	337.11 ± 14.84	-	-	76.96 ± 5.24	34.40 ± 2.77	18.19 ± 0.93	0.15 ± 0.02
8	332.87 ± 12.04	-	-	75.96 ± 2.42	33.70 ± 1.64	17.85 ± 1.11	0.14 ± 0.02
16	328.63 ± 7.10	-	-	72.48 ± 1.02	31.82 ± 1.94	14.03 ± 0.45	0.11 ± 0.01
24	201.68 ± 11.43	-	-	66.92 ± 0.47	28.57 ± 0.37	12.71 ± 0.32	0.11 ± 0.02
**UVC Exposure**	**Mechanical Parameter–Tensile Test of PBS-CB**						
**Time** **(h)**	**E** **[MPa]**	**σ_y_** **[MPa]**	**ε_y_** **[%]**	**Load r** **[N]**	**σ_r_** **[MPa]**	**ε** **_r_** **[%]**	**L_r_** **[J]**
0	449.20 ± 17.23	43.86 ± 1.26	20.82 ± 1.69	85.41 ± 3.47	52.28 ± 5.12	470.48 ± 27.55	4.64 ± 1.34
2	301.19 ± 16.97	36.48 ± 1.30	21.33 ± 1.16	93.71 ± 14.23	38.71 ± 4.10	387.54 ± 18.79	4.15 ± 0.74
4	294.94 ± 19.30	34.13 ± 0.49	22.22 ± 1.07	87.59 ± 10.00	35.12 ± 4.31	345.27 ± 30.12	3.96 ± 1.02
8	277.55 ± 21.24	33.86 ± 3.22	19.41 ± 1.16	83.88 ± 14.89	33.81 ± 2.26	336.82 ± 15.78	3.78 ± 0.42
16	272.41 ± 26.46	33.75 ± 0.83	18.39 ± 1.56	74.63 ± 2.49	31.77 ± 1.93	307.90 ± 22.19	3.22 ± 0.85
24	264.02 ± 23.11	28.72 ± 2.21	21.82 ± 0.41	71.38 ± 3.85	28.33 ± 2.59	304.05 ± 11.48	2.83 ± 0.67

**Table 3:** Crystalline degree values of PBS and PBS-CB during the UVC exposure time.

UVC Rays	Melting Enthalpy		Melting Temperature		Crystalline Degree	
Exposure Time (h)	PBS ΔH_m_ (J/g)	PBS-CB ΔH_m_ (J/g)	PBS (°C)	PBS-CB (°C)	PBS (%)	PBS-CB (%)
0	74.75 ± 0.40	80.62 ± 0.71	118.73 ± 0.32	119.63 ± 0.24	67.76 ± 0.65	74.54 ± 0.46
2	74.62 ± 0.04	72.32 ± 0.54	118.29 ± 0.24	118.79 ± 0.68	64.25 ± 0.34	66.92 ± 0.73
4	60.16 ± 0.46	83.66 ± 0.71	118.22 ± 0.53	118.73 ± 0.43	54.57 ± 0.42	58.98 ± 0.56
8	63.37 ± 0.45	63.91 ± 0.34	117.41 ± 0.19	117.55 ± 0.39	57.46 ± 0.37	59.09 ± 0.39
16	81.44 ± 0.28	57.32 ± 0.29	117.06 ± 0.89	117.23 ± 0.27	73.85 ± 0.85	53.04 ± 0.53
24	78.71 ± 0.38	57.17 ± 0.71	116.60 ± 0.12	116.72 ± 0.64	71.34 ± 0.64	52.93 ± 0.55

**Table 4:** Wettability of distilled water and blood on PBS and PBS-CB.

UVC Exposure	Wenzel Contact Angle, (*θ_w_*) [°]			
	PBS		PBS-CB	
Time (h)	Distilled Water	Blood	Distilled Water	Blood
0	82.2 ± 0.95	82.1 ± 1.02	91.1 ± 1.53	73.9 ± 0.99
2	79.7 ± 1.14	75.8 ± 1.22	80.5 ± 1.38	71.8 ± 1.28
4	77.8 ± 1.10	72.5 ± 1.11	78.4 ± 1.62	69.5 ± 1.09
8	76.2 ± 1.04	69.3 ± 0.96	77.4 ± 1.33	67.8 ± 0.86
16	75.5 ± 0.98	68.4 ± 0.77	76.3 ± 1.66	63.2 ± 0.96
24	74.6 ± 1.13	67.0 ± 1.18	75.2 ± 1.34	62.8 ± 1.00

**Table 5:** Operative parameters of 254 nm UVC irradiation of polymers employed in the literature papers.

Refs.	Power (W)	Intensity (10^−3^ W/cm^2^)	Fluence Dose (J/cm^2^)	Time (min)	Distance Sample–Lamp (cm)	Polymer/Killed Microorganisms (%)
[34]	n.a. *	1.0	20 × 10^−3^	1440	n.a. *	ABS-PC/90
[48]	6	0.4	2.16	90	9	Silicone/98.7
			1.4 × 10^3^	60	9	Silicone/99.9
[49]	n.a. *	3.12	n.a. *	120–960	5	PLA/n.a. *
[50]	30	0.125	2.24 × 10^−4^	30	100	n.a. */90
[51]	18	10.12	729 × 10^−3^	2	17	PMMA-ABS-PU/99.99
This work	6	0.86	6.2–74.3	120–1440	15	PBS/n.a. *

* not available.

## Data Availability

The data presented in this study are available on request from the corresponding author.
